# Comparison of static and dynamic exposures to air pollution, noise, and greenness among seniors living in compact-city environments

**DOI:** 10.1186/s12942-023-00325-8

**Published:** 2023-01-28

**Authors:** Oriol Marquet, Jose Tello-Barsocchini, Daniel Couto-Trigo, Irene Gómez-Varo, Monika Maciejewska

**Affiliations:** 1 Institute of Environmental Science and Technology (ICTA‐UAB), Universitat Autònoma de Barcelona, 08193 Cerdanyola del Vallès, Barcelona Spain; 2 Research Group on Mobility, Transportation and Territory (GEMOTT), Department of Geography, Universitat Autònoma de Barcelona, 08193 Cerdanyola del Vallès, Barcelona Spain; 3 Cosmopolis Centre for Urban Research, Vrije Universiteit Brussels, Brussels, Belgium

**Keywords:** Exposure assessment, Air pollution, Greenness, Noise, Older adults, Dynamic exposure

## Abstract

GPS technology and tracking study designs have gained popularity as a tool to go beyond the limitations of static exposure assessments based on the subject's residence. These dynamic exposure assessment methods offer high potential upside in terms of accuracy but also disadvantages in terms of cost, sample sizes, and types of data generated. Because of that, with our study we aim to understand in which cases researchers need to use GPS-based methods to guarantee the necessary accuracy in exposure assessment. With a sample of 113 seniors living in Barcelona (Spain) we compare their estimated daily exposures to air pollution (PM2.5, PM10, NO2), noise (dB), and greenness (NDVI) using static and dynamic exposure assessment techniques. Results indicate that significant differences between static and dynamic exposure assessments are only present in selected exposures, and would thus suggest that static assessments using the place of residence would provide accurate-enough values across a number of exposures in the case of seniors. Our models for Barcelona’s seniors suggest that dynamic exposure would only be required in the case of exposure to smaller particulate matter (PM2.5) and exposure to noise levels. The study signals to the need to consider both the mobility patterns and the built environment context when deciding between static or dynamic measures of exposure assessment.

**Supplementary Information:**

The online version contains supplementary material available at 10.1186/s12942-023-00325-8.

## Highlights

- Static and dynamic exposures were estimated for a set of basic exposures (NO2, PM2.5, PM10, dB, NDVI) among 113 seniors in Barcelona.
- Static exposures were based on residential location while 7-day GPS tracking was used for dynamic exposures.
- Dynamic assessments using GPS-tracking are not providing enough accuracy across all the range of exposures.
- PM2.5, and noise exposures significantly benefited from GPS-tracking use.
- NO2, PM10, and exposures to greenness did not lose significant accuracy using only residential static exposures with Barcelona’s seniors

**Supplementary Information:**

The online version contains supplementary material available at 10.1186/s12942-023-00325-8.

## Introduction

Research on how environmental exposures directly affect human health has gained attention in recent years. The assessment of how exposure to different environmental features can harm or promote health has greatly benefitted from more accurate data both on the spatial distribution of exposures and on human positioning and travel behavior. To date, available scientific evidence has amply demonstrated the associations between daily average exposures to factors such as air pollution, noise, and greenness and cardiovascular disease, mental health, and even wellbeing [1–3].

### Static vs dynamic exposure assessment

Traditionally, most environmental exposure assessments have taken the residence location or the workplace as a proxy for people’s environmental exposures [4, 5]. Using geolocated addresses has been a major step forward from using just the administrative neighborhood [6]. However, in real life, most people move beyond their residential areas during their everyday travel, and thus static residential neighborhoods cannot capture the entire context of exposure [7–9]. Some have suggested that the literature needs to move beyond notions of contextual influence that rely on using such specific fixed locations [10, 11]. GPS and tracking technologies have been viewed as the optimal solution to go beyond where people live to where people visit and how much time they spend at each particular location [12, 13]. GPS location and tracking trajectories can accurately identify people’s space–time trajectories, frequency, and duration which can provide dynamic measures of exposure measures that stand in contrast to traditional static address-based measures. These dynamic measures of exposure can greatly improve the accuracy of exposure assessments either at the momentary level or at the activity space daily aggregate level [14–16].

Dynamic assessments of exposures also allow for avoiding commonly identified limitations of neighborhood effects research, such as the uncertain geographic context problem (UGCoP) [17] or the neighborhood effect averaging problem (NEAP) [18]. Such approaches, however, are far more resource-intensive, require more specific research designs [19], and may be affected by other common spatial biases such as the selective daily mobility bias [20]. Most importantly, studies using raw GPS data usually need to use additional methods to add contextual information on individuals’ time-activity patterns [21] and they require intensive engagement from study participants which usually leads to small sample groups, vulnerable to participation rates, and study-abandoning [22]. Participants in studies requiring location tracking and high spatio-temporal precision also report concerns with data privacy and surveillance. Because of that, it is important to understand when it is necessary to use dynamic exposures and in which situations we can expect high-accuracy exposure assessments from static measures alone. Deciding between static or dynamic exposure measurements is a complex task that will likely depend on the subject of analysis and the type of exposure of interest.

This need is even more important when dealing with specific population groups such as seniors or children for whom wearing a specific tracking device may be more difficult than the adult population. In addition, real exposures have been found to vary widely between social groups. That is mainly because of differences in location patterns and daily mobility habits. Previous research has found socioeconomically disadvantaged groups to be more frequently in contact with hazardous exposures [8]. Because of the link between travel and exposure, sociodemographic groups with different travel patterns are likely also to differ in their daily exposures. General assessments of exposure to the overall population are, for example, not often applicable to seniors due to their distinct spatial practices and travel behavior.

While studying seniors’ exposure levels to air pollutants, noise or greenness is particularly important because of their larger chronic diseases’ prevalence [23, 24], these assessments often require specific study designs given seniors’ special relationship with their neighborhood and their most near built environment [25]. Seniors spend higher amounts of time in the close vicinity of their residences [26–28] which would suggest that residential-based exposure assessments would provide accurate-enough exposure measures, but at the same time, they also tend to engage in higher amounts of walking trips [29] which increases their sensitivity to environmental exposures. While the consensus seems to be that seniors tend to have smaller and more compact activity spaces [30, 31] they cannot be considered home-bound, as their daily mobility is complex and they show great variance in their activity spaces sizes and shapes [32]. Previous studies have found that given the proper built environment conditions, seniors can significantly extend their living spaces and even spend more time outside of their residential neighborhood than inside of it [33, 34]. That is especially true in walkable and compact cities such as Barcelona where seniors most often used modes of transport -walking and public transport- can take them far within the city.

### Common environmental exposure assessments

Choosing between static or dynamic exposure assessment methods, however, will not only be dependent on the population group, but also the nature of the exposures. Studies comparing the spatial distribution of environmental exposures such as carbon dioxide (CO2), Fine particulate matter PM2.5 and PM10, nitrogen dioxide (NO2), or sulfur dioxide (SO2) have found little to no spatial correlation between them [35–37] suggesting that the decision on whether to use dynamic or static measurements would have to be also exposure dependent. To date, studies estimating the effects of environmental exposures on seniors have focused on air pollution, noise pollution, and greenness [38–41].

Studies estimating the effects of air pollution typically use a combination of NO2, PM10, and PM25 as air quality indicators. These pollutants are mainly emitted by internal combustion vehicles and are the most prevalent air pollutants in urban environments [42, 43], including in Barcelona [44, 45]. Their public health dangers are well documented [46] and seniors are considered one of the most at-risk populations [47]. Ambient particulate matter is recognized as one of the main environmental risk factors for chronic respiratory diseases [48, 49] and cognitive decline among them [50, 51].

Exposure to unwanted sound from industry, transport, or other urban activities is commonly referred to as noise pollution [52–54]. Noise pollution is a public health issue that has been is gaining prominence as scientific evidence linking noise and health increases [55]. Among these links, studies have found noise to be associated with sleep disturbances, hypertension, cardiovascular risks, chronic stress, or disturbances in mental health [56, 57]. Recent analyses have also found seniors to be more affected by noise than the rest of the population even when exposed to a similar level of measured noise [58]. Most worrisome, seniors also tend to live in areas with higher concentrations of noise pollution [59] something that Lagonigro et al. [60] also found in Barcelona.

However, there are also positive environmental exposures for seniors, such as exposure to greenness. Being in contact with nature, which includes having visual access to green space, has been associated with a wide range of positive health effects, from physical to mental health and restorative processes [61–64]. Urban greenness, commonly measured using the Normalized Difference Vegetation Index (NDVI) [61, 62, 65–67] can provide optimal places to walk or recreate while also being negatively correlated with other negative urban exposures such as air or noise pollution. Among seniors, exposure to green space has been linked to many health outcomes, including mortality, social capital, obesity, and most frequently with physical activity [68–70].

Given the high number of biases that affect neighborhood effects research, more light is needed to define what is the appropriate scale at which to measure exposure among specific population groups. Considering this need for a better understanding of when to use dynamic vs static research designs in studies involving the senior population, this study sets to estimate exposure to air pollutants, noise, and greenness in a sample of 113 older people living in Barcelona and participating in a GPS-tracking study for 7 days. The study sets out to answer whether using static exposure techniques based on the residential address is enough to represent the totality of exposures experienced throughout a day. That is to understand if continuing to use static exposure measurements instead of dynamic-based ones will compromise the reliability of exposure assessment studies among seniors.

## Methods

This study was set in the context of the RecerCaixa Project (“Ciudad, calidad de vida y movilidad activa en la tercera edad. Un análisis multi-metodológico a través de Tracking Living Labs”) and took place in the municipality of Barcelona. The project aimed to explore basic mobility patterns of seniors (> 64 years old) living in the Barcelona metropolitan area along with quantifying the environmental, social and health issues that impact their daily mobility choices.

### Study design and population sample

With more than one-fifth of its population being over 65 years old, Barcelona is usually defined as a compact, walkable, and vital city [71–74]. Barcelona’s morphological conditions are representative of other historical Mediterranean cities, combining high population density and land use mix that create walkable environments but also maintaining high car-use levels due to extensive motorization rates along with significant car-dependency in parts of the metropolitan area [75]. Due to its density and high vehicle use, Barcelona has also high average levels of air pollution and noise pollution [76–78]. Despite having a low ratio of green-space per inhabitant compared to other European cities (18 m^2^ including the peri-urban forest of Collserola), small parks, streets, boulevards, and plazas with trees often compensate for the need for spaces for outdoor activities and provide an optimal distribution of NDVI (Normalized Difference Vegetation Index) levels [79, 80].

Participants were recruited between June 2016 and June 2017. Researchers contacted 39 senior centers scattered through the Barcelona metropolitan area to recruit seniors to participate in a tracking-GPS data gathering. Participants had to be 65 years old or above and not have specific mobility impairments. After being informed written and orally about the study, and provided with research protocols and instructions, 269 participants gave informed consent, out of those, we focused on those that lived within the limits of the Barcelona municipality (n = 113). Confidentiality was ensured by using random identification numbers and data censoring. The study was approved by the Ethics Committee on Animal and Human Experimentation at Universitat Autònoma de Barcelona (UAB; CEEAH-3656). Considering that seniors’ outdoor behavior in relation to the built environment presents differences according to their characteristics, we classified our sample by age [younger (< 75 years old), and older (≥ 75 years old) seniors] and gender.

### Data collection

To collect data regarding the routine travel behavior of seniors, participants were asked to wear a GPS device (QStarz BT-Q1000X; QStarz International Co., Ltd., Taiwan, R.O.C.) and a wrist-worn accelerometer (Actigraph GT3X + ; ActiGraph LLC, Pensacola, Florida USA) for seven consecutive days. Valid days included at least four wearing days and ten hours of device wear-time. Participants were also asked to fill in a questionnaire disclosing their age, gender, self-reported health, and perceived characteristics of their residential neighborhood. The Physical Activity Location Measurement System (PALMS) v.R4 was used to aggregate data extracted from GPS and accelerometer devices into 15 s intervals [16].

**Fig. 1:**
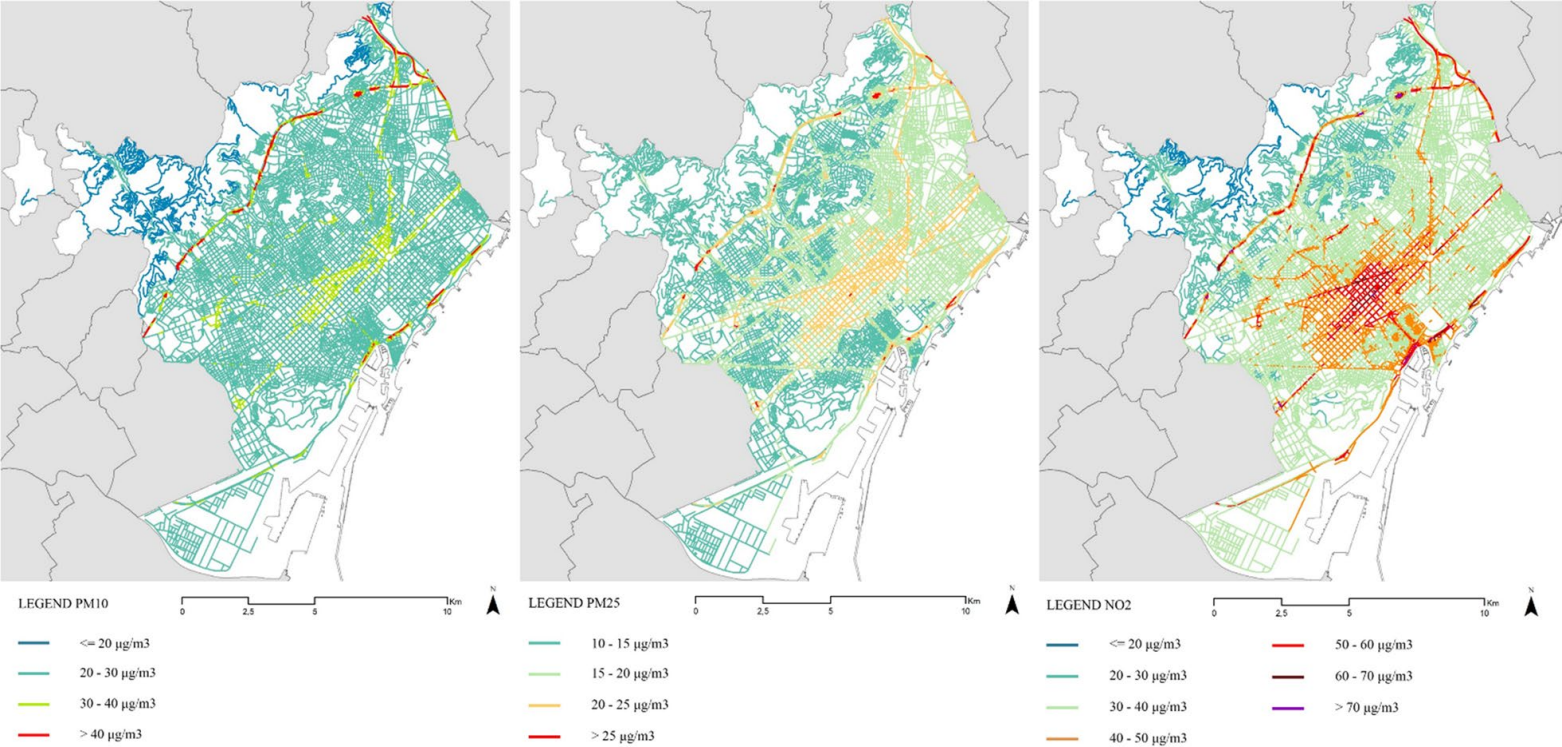
Daily air pollution levels spatial distribution in Barcelona: PM10 (left), PM2.5 (center), NO2 (right). Source: Ajuntament de Barcelona

**Fig. 2:**
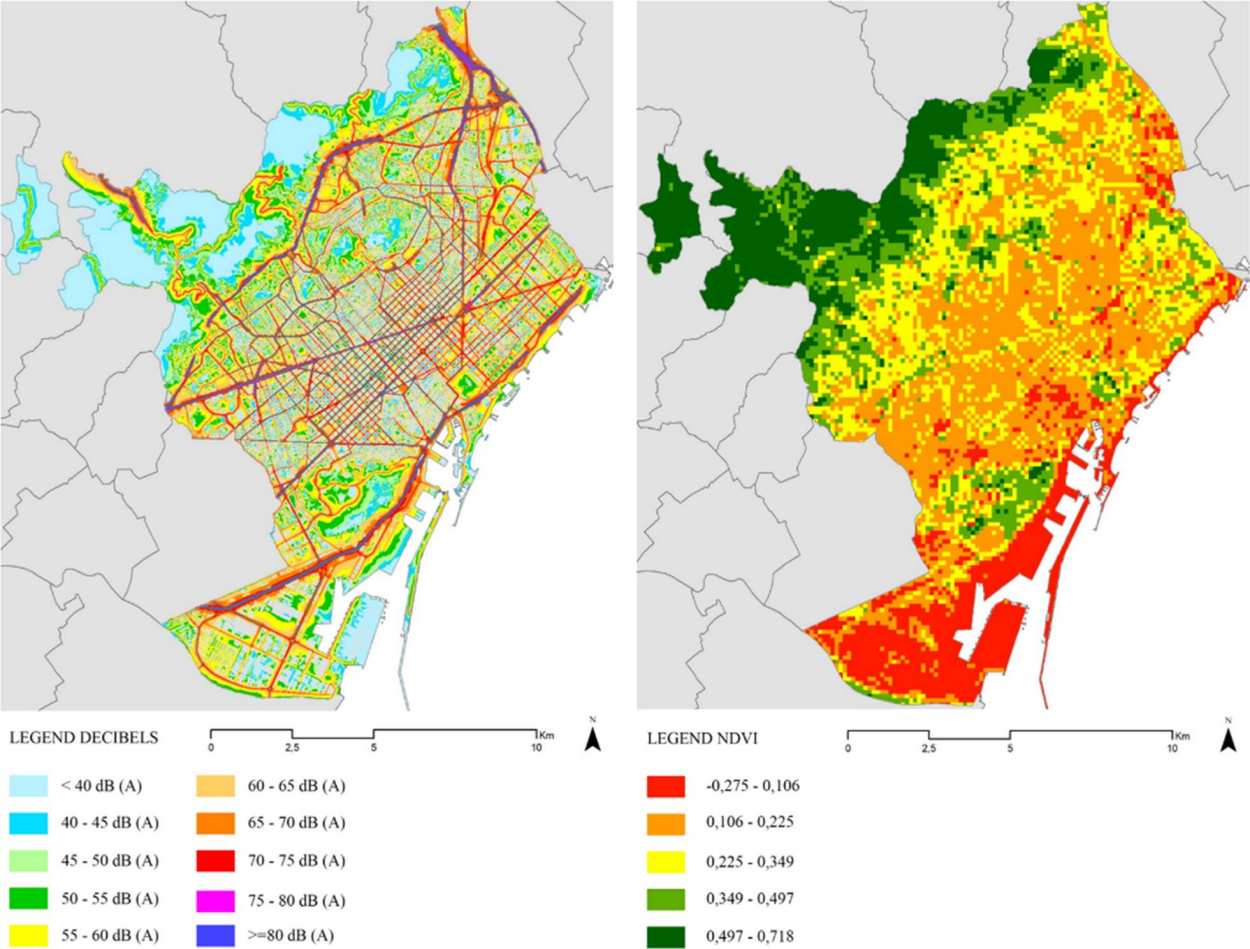
Daily noise levels (left) and greenness (right) spatial distribution in Barcelona

Spatial exposure to air quality components was based on the air quality inmission maps provided by the Barcelona municipality with data from 2019 (Fig. 1). These maps provide data on nitrogen dioxide (NO2), and suspended particulates PM10 and PM2.5 based on annual averages and are calculated at the street section level. The spatial modeling of the dispersion of pollutants is performed by the municipality and offers a complete map of average immission levels at the street-section scale (Fig. 2L). Similar to Chum and Ocampo [81] and Nyhan et al. [12] we used average daily NO2, PM10, and PM2.5 concentrations as a proxy of exact exposures.

Data on noise exposure were based on the noise contour maps from the strategic noise map of the city of Barcelona, obtained through the Barcelona Open Data portal with data from 2017 (Fig. 2). These are the result of the collaboration between the Barcelona city council and the Barcelona Public Health Agency (ASPB) and represent noise levels on a daily average (7 am to 9 pm). The spatial distribution of noise exposure is represented in Fig. 2 (left).

Finally, we used NDVI to represent the levels of urban greenness in Barcelona. Due to healthy green vegetation reflecting more infrared radiation and absorbing more energy in the red wavelength compared to unhealthy vegetation or surfaces without vegetation, NDVI is commonly used to assess vegetation levels. The NDVI database of Catalonia 2019 available on the portal of the Cartographic and Geological Institute of Catalonia [82] was used. NDVI orthoimages are generated from images obtained by an aerial photogrammetric camera, capable of obtaining information not only from the visible range of the electromagnetic spectrum but also from the near-infrared, and with a pixel size of about 25.cm on the ground. NDVI scores can vary from -1 to 1 when the result is < 0 corresponds to areas without vegetation cover corresponding to water or artificial surfaces, for values 0 < NDVI < 0.2 it is expected to find bare soil or dead vegetation, between 0.2 < NDVI < 0, 4 corresponds to soil with sparse or not very vigorous vegetation, in cases of 0.4 < NDVI < 0.6, areas with vigorous and abundant vegetation are estimated, while those exceeding < 0.6 correspond to areas with dense and vigorous vegetation [82]. NDVI use is very common in studies assessing greenness exposure [83–86] and has also been used before in Barcelona [61, 62]. The distribution of NDVI levels in Barcelona is presented in Fig. 2 (right).

The spatial resolution of environmental exposures maps allowed us to pair average environmental conditions to GPS points using GIS spatial analysis techniques. These allowed us to assess the variability of intensity of exposures based on spatial location and movements of seniors. It was, however, not possible to assess temporal variability as environmental exposures represented daily averages and were thus not disaggregated by hour or time of day.

A series of covariates were extracted from the participants baseline survey such as gender and age (mean = 74.9; sd = 7.56). Based on their place of residence we also calculated their income and population density based on census track data. In order to ease interpretation, we also grouped seniors per types of built environment with historic district representing the older parts of the city, Expansion representing the compact development starting around 1850 and sprawl areas representing the more recent development areas characterized by low densities and high car dependency.

### GIS processing

**Fig. 3:**
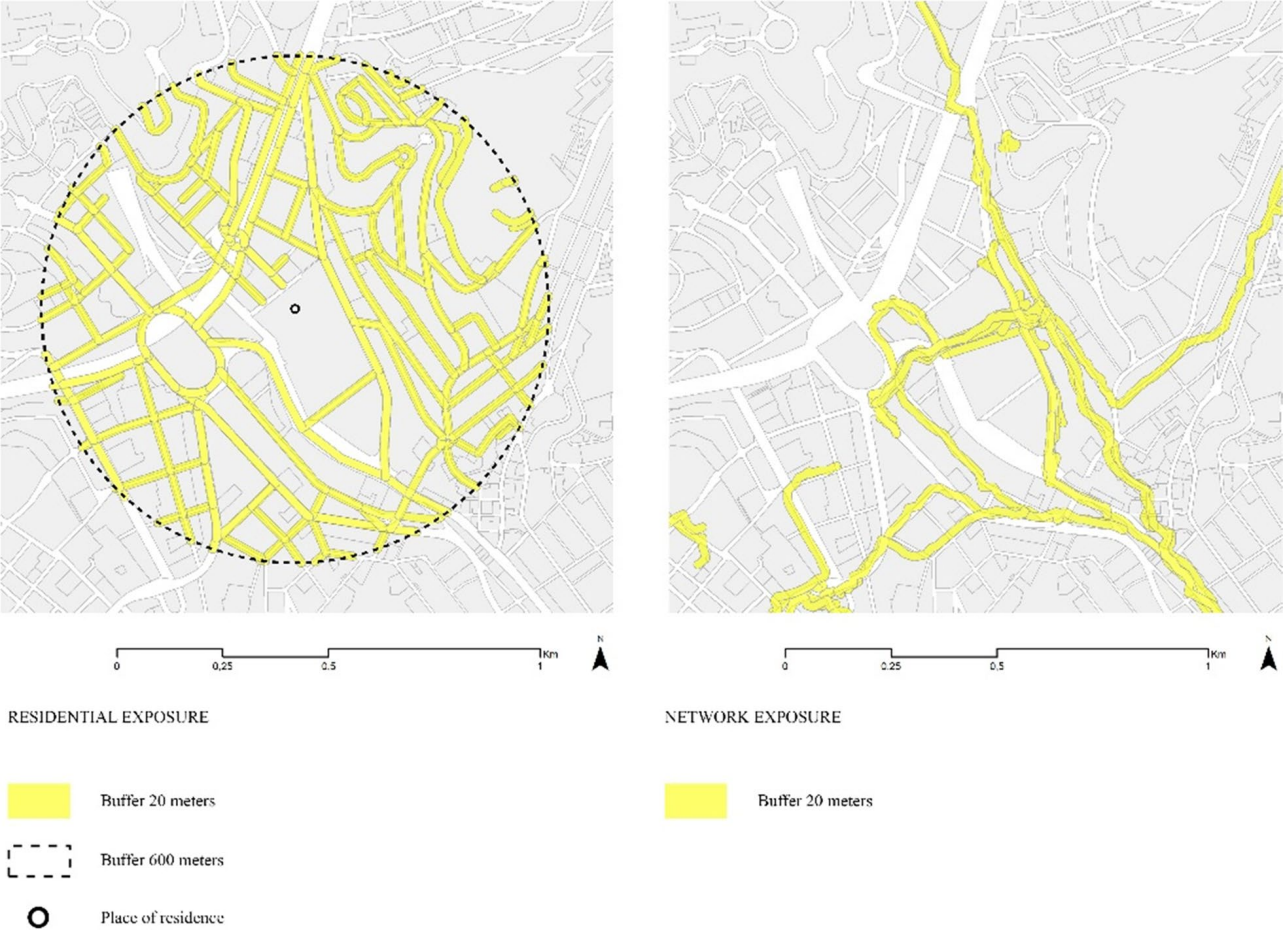
Residential (left) and dynamic (right) exposure buffers of one day of data collection

Using the self-reported home address of each participant we created a street network buffer of 600 m. Within these 600 m, the second buffer of 20 m was estimated, calculated by including only those roads that are walkable. Thus, the residential area of exposure is the result of a network buffer of 20 m around the roads where walking is possible and that falls within 600 m from the place of residence. to define the residential extent of exposures (Fig. 3, left). The 600-m buffer was estimated based on previous studies on older adult mobility [71, 87, 88] and corresponds to approximately 10 min of walking for that age group which has been a walkable distance for the population group. The 20-m buffer corresponds to the average street width in Barcelona [79]. To capture the range of dynamic exposure we used a 20-m buffer around all walking tracks accumulated by each participant throughout each participating day (Fig. 3, right) following [5, 14]. We chose a 20-m buffer because the GPS accuracy cannot distinguish which side of the street the participant is using and thus covering the whole street width was deemed necessary.

**Fig. 4:**
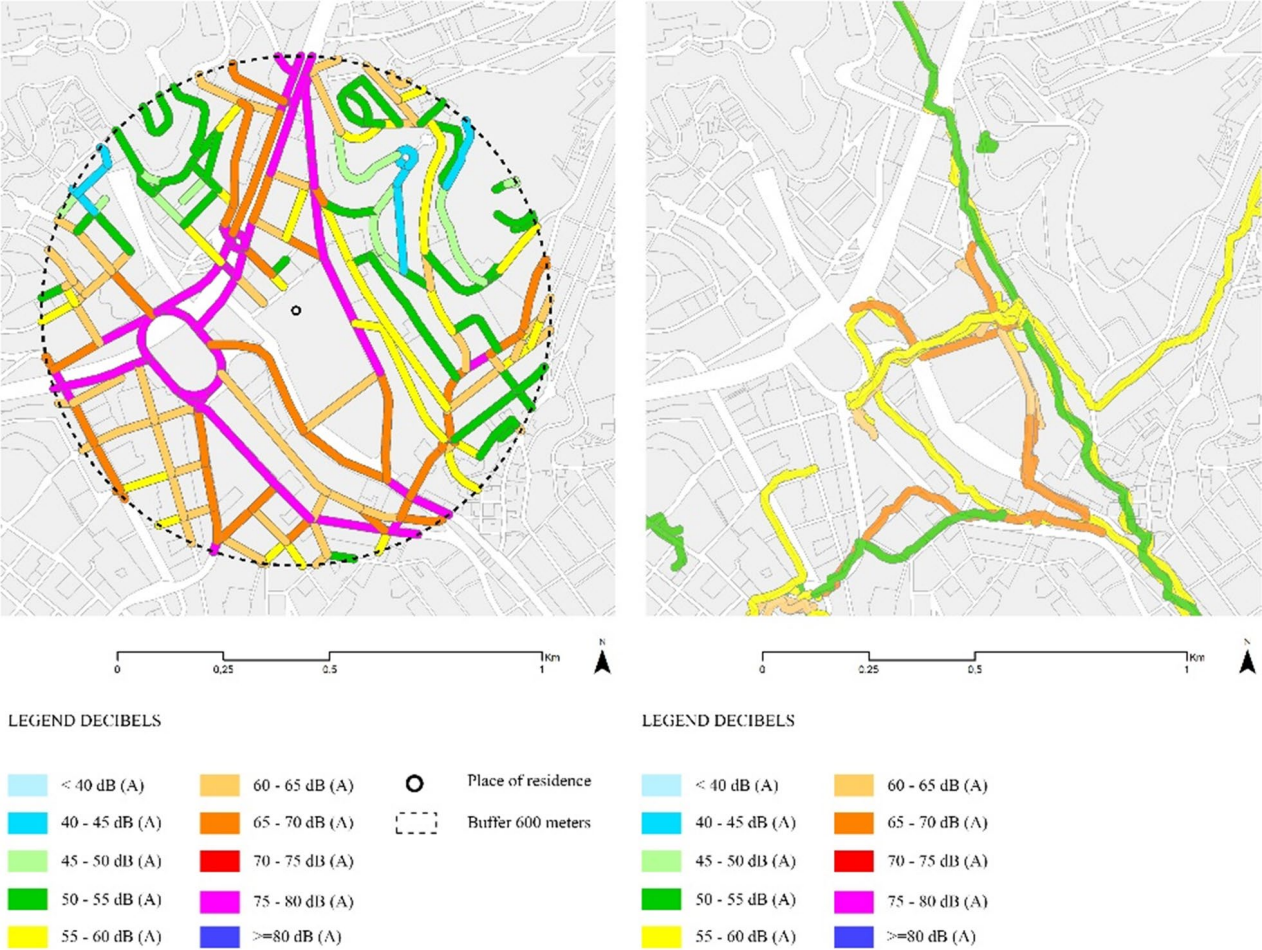
Example of noise exposure calculation based on residential (left) and dynamic (right) buffers

Once residential and dynamic exposure ranges were defined, we used spatial join to average the exposures within each buffer of 600 m, thus creating daily average exposures for NO2, PM10, PM2.5, noise, and NDVI respectively (Fig. 4). All processes were conducted using ArcMap 10.7.1.

### Statistical analysis

Using daily averages of exposure, we tested the differences between home and dynamic average daily exposures. Pairwise correlations between exposures are available in Additional file 1: Table S1. To do so we used paired sample t-test to examine home-dynamic averages that were significantly different from each other. NO2, PM10, PM2.5, noise, and NDVI daily average exposure measurements were compared using t-tests to assess the overall level of agreement. To visualize exposure differences, we used boxplots while stratifying the results by age group and gender of the participants.

To examine the role of any sociodemographic or contextual variable driving the differences in exposure, we used a 5 model multivariable analysis in which we use multilevel Poisson models estimating the differences between residential and dynamic environmental exposures. Each model uses the difference between home and dynamic exposure levels for each environmental exposure type (model 1: PM10; model 2: PM2.5; model 3: NO2; model 4: dB; model 5: NDVI). Each model estimates the effect of gender and age of the participant and the urban layout and average income level of the participant’s home neighborhood while also controlling for the number of walking trips conducted each day. To account for the nested nature of our data, we employ multilevel models using the participant’s ID as a random effect. All analysis were run using Stata v16.

## Results

Average exposure levels for the main three air quality pollutants -PM10, PM2.5, NO2-, are shown in Table 1. Differences between home and dynamic exposure are tested using paired samples t-tests.

**Table 1:** Home and dynamic daily exposures to PM10, PM2.5, and NO2 per population group

	PM10				PM2.5				NO2			
	Home^a^	Dynamic^b^	Diff. %^c^	*p*^d^	Home^a^	Dynamic^b^	Diff. %^c^	*p*^d^	Home^a^	Dynamic^b^	Diff. %^c^	*p*^d^
Total (N = 113)	25.64	25.90	1.00	0.006	16.17	17.16	5.77	0.000	37.55	38.05	1.33	0.036
Sex												
Men (N = 55)	25.71	26.17	1.74	0.001	16.27	17.21	5.47	0.000	37.23	37.91	1.77	0.022
Women (N = 58)	25.57	25.64	0.29	0.559	16.07	17.11	6.05	0.000	37.84	38.19	0.92	0.357
Age												
65 to 74 y.o. (N = 58)	25.85	25.89	0.14	0.808	16.17	17.07	5.25	0.000	37.93	38.29	0.96	0.315
75 + y.o. (N = 55)	25.41	25.91	1.92	0.000	16.16	17.25	6.32	0.000	37.14	37.79	1.73	0.036
Urban layout												
Expansion (N = 91)	25.80	26.20	1.53	0.000	16.50	17.50	5.69	0.000	38.05	38.60	1.41	0.042
Sprawl (N = 6)	23.69	22.48	− 5.37	0.259	14.16	15.20	6.81	0.022	31.20	31.41	0.67	0.838
Historic District (N = 16)	25.43	25.42	− 0.03	0.923	15.01	15.94	5.89	0.000	36.98	37.39	1.09	0.227
Income												
High (N = 68)	25.75	26.05	1.15	0.000	15.68	16.52	5.13	0.000	39.08	39.37	0.76	0.384
Low (N = 45)	25.48	25.68	0.79	0.173	16.88	18.08	6.61	0.000	35.33	36.14	2.24	0.001
Density												
High (N = 107)	25.74	26.08	1.30	0.000	16.28	17.26	5.72	0.000	37.89	38.41	1.36	0.025
Low (N = 6)	23.69	22.48	− 5.37	0.259	14.16	15.20	6.81	0.022	31.20	31.41	0.67	0.838

^a^Residence-based exposure measured on a 600 m street-network buffer from the geocoded participant’s address

^b^Dynamic-based exposure measured on walking Daily Path Areas

^c^Difference between Home and Dynamic exposures as a percentage of home exposure

^d^Paired samples t-test

In terms of PM10, the average participant was exposed in a single day to areas with 25.64 µg/m^3^ annual immission levels, while their dynamic exposure was 25.9 µg/m^3^. While a statistically significant difference, the estimated value using GPS tracking was only 1.0% different than the estimated value using the home exposure (*p* = 0.006). When stratified by population groups, the larger statistically significant differences between home and dynamic PM10 exposure were found in those participants that were older than 75 years old, who registered dynamic exposures 1.92% higher than home exposure (*p* < 0.001). Other statistically significant differences were found among men (1.74%; *p* < 0.001), those living in the Expansion area of the city (1.53%; *p* < 0.001), and those living in high-income areas (1.15%; *p* < 0.001), although no difference was greater than 2%.

In terms of exposure to PM2.5, the average daily exposure difference was 5.77% higher when calculated using dynamic GPS tracking than using the home method (*p* < 0.001). All population groups registered statistically significant differences, with no differences below 5%. The maximum differences were found among women (6.05%; *p* < 0.001), the older population (6.32%; *p* < 0.001), and those living in sprawl or low-density areas (6.81%; *p* = 0.022).

The NO2 exposure assessment registered a daily exposure difference between the home and dynamic method of 1.33 on average (1.33%; *p* = 0.036). The only statistically significant differences were found among men (1.77%; *p* = 0.022), the older population (1.73%; *p* = 0.036), those living in the Expansion (1,41%; *p* = 0.042) and most significantly in low-income areas (2.24%; *p* = 0.001).

Average exposure levels for noise (dB) and greenness (NDVI) are shown in Table 2. In terms of noise, the average participant lived in an area with an average of 58.6 daily db. In contrast, the average participant was exposed to areas with 64.6db during his/her daily walking trips. The estimated dynamic exposure was thus 9.35% lower than the home exposure (*p* < 0.001). Almost all population groups share that same difference between 9 and 11%. The larger differences are found among those who live in the Expansion area (− 11.06; *p* < 0.001) or the historic district (− 11.1; *p* < 0.001) and among those living in high-income areas (− 11; *p* < 0.001).

**Table 2:** Home and dynamic daily exposures to noise (dB) and greenness (NDVI) per population group

	dB				NDVI			
	Home^a^	Dynamic^b^	Diff. %^c^	*p*^d^	Home^a^	Dynamic^b^	Diff. %^c^	*p*^d^
Total (N = 113)	58.66	64.62	− 9.35	0.000	0.237	0.24	0.44	0.708
Sex								
Men (N = 55)	58.61	64.42	− 9.03	0.000	0.236	0.23	− 1.53	0.340
Women (N = 58)	58.54	68.40	− 9.66	0.000	0.238	0.24	2.21	0.189
Age								
65 to 74 y.o. (N = 58)	65.37	59.29	− 10.26	0.000	0.225	0.235	− 0.23	0.875
75 + y.o. (N = 55)	63.81	57.80	− 10.39	0.000	0.249	0.25	1.07	0.539
Urban layout								
Expansion (N = 91)	65.48	58.60	− 11.06	0.000	0.239	0.24	0.47	0.697
Sprawl (N = 6)	58.92	57.50	− 2.48	0.197	0.336	0.31	− 8.92	0.002
Historic District (N = 16)	65.48	58.96	− 11.1	0.000	0.190	0.20	5.44	0.004
Income								
High (N = 68)	66.12	59.56	− 11.00	0.000	0.216	0.21	− 1.25	0.272
Low (N = 45)	62.45	57.14	− 9.29	0.000	0.268	0.27	2.33	0.160
Density								
High (N = 107)	64.93	58.63	− 10.74	0.000	0.232	0.23	15.48	0.330
Low (N = 6)	58.93	57.5	− 2.48	0.197	0.336	0.31	− 8.92	0.002

^a^Residence-based exposure measured on a 600 m street-network buffer from the geocoded participant’s address

^b^Dynamic-based exposure measured on walking Daily Path Areas

^c^Difference between Home and Dynamic exposures as a percentage of home exposure

^d^Paired samples t-test

Greenness for its part did not show major significant differences when assessed using the home range or the dynamic exposure (0.44%; *p* = 0.708). Only those living in the historic district of the city recorded a 5% difference between the home and the dynamic exposure, with the dynamic exposure being higher (home = 0.190 vs dynamic = 0.201; *p* = 0.004).

**Fig. 5:**
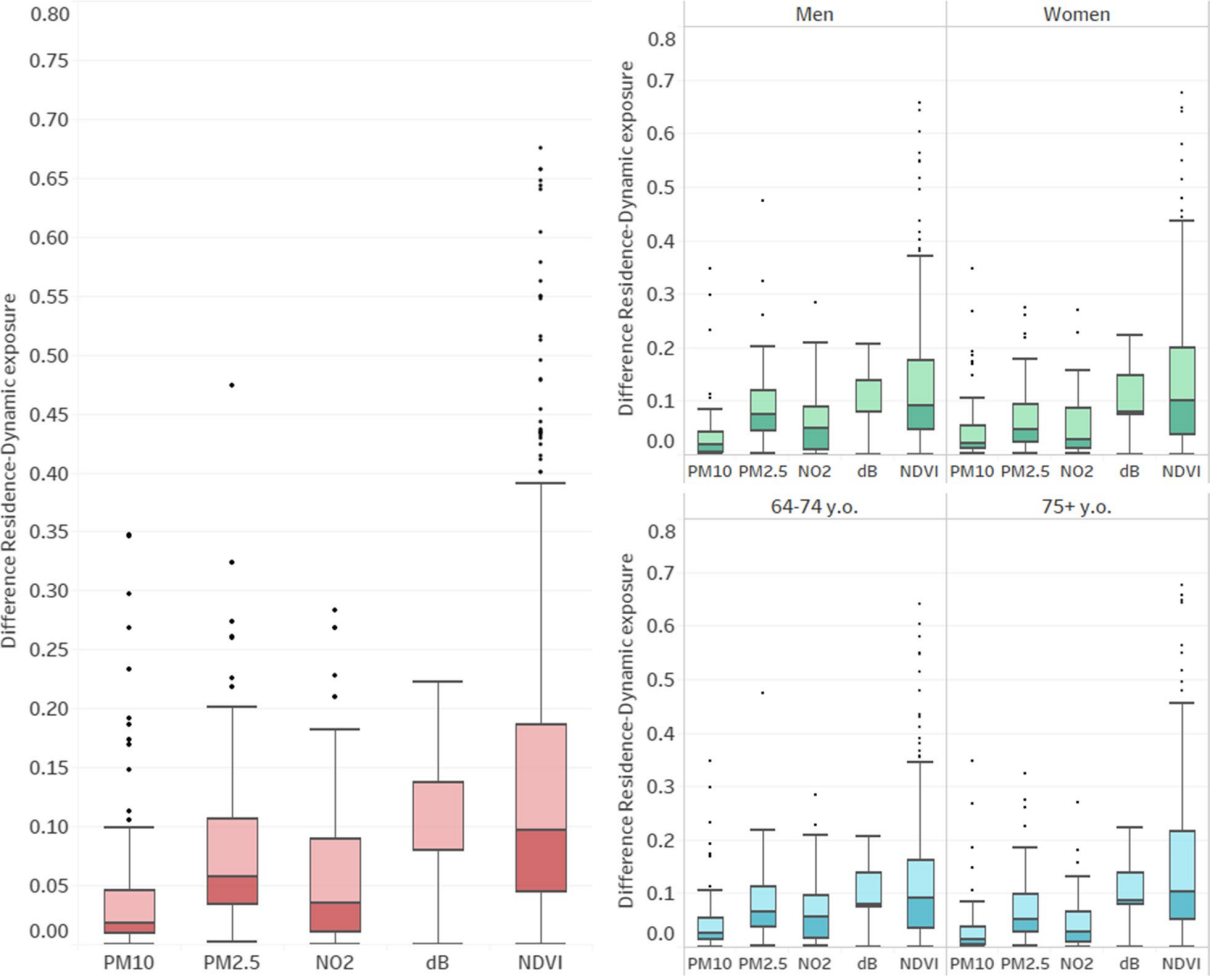
Distribution boxplots for differences between residence and dynamic exposure per gender, age When we interact the differences by gender and age (Fig. 6) we can spot similar differences with very little changes in the means but with interquartile ranges being consistently wider in the case of males, especially in the case of NDVI

The graphical representation of the differences between residence and dynamic exposures (Fig. 5) shows there are higher mean differences in the case of PM2.5, noise, and NDVI. When focusing on gender and age, women and those older than 75 portray higher mean differences in noise and NDVI, while men and elders' younger than 75 show higher mean differences in PM2.5 and NO2, thus in air pollution.

**Fig. 6:**
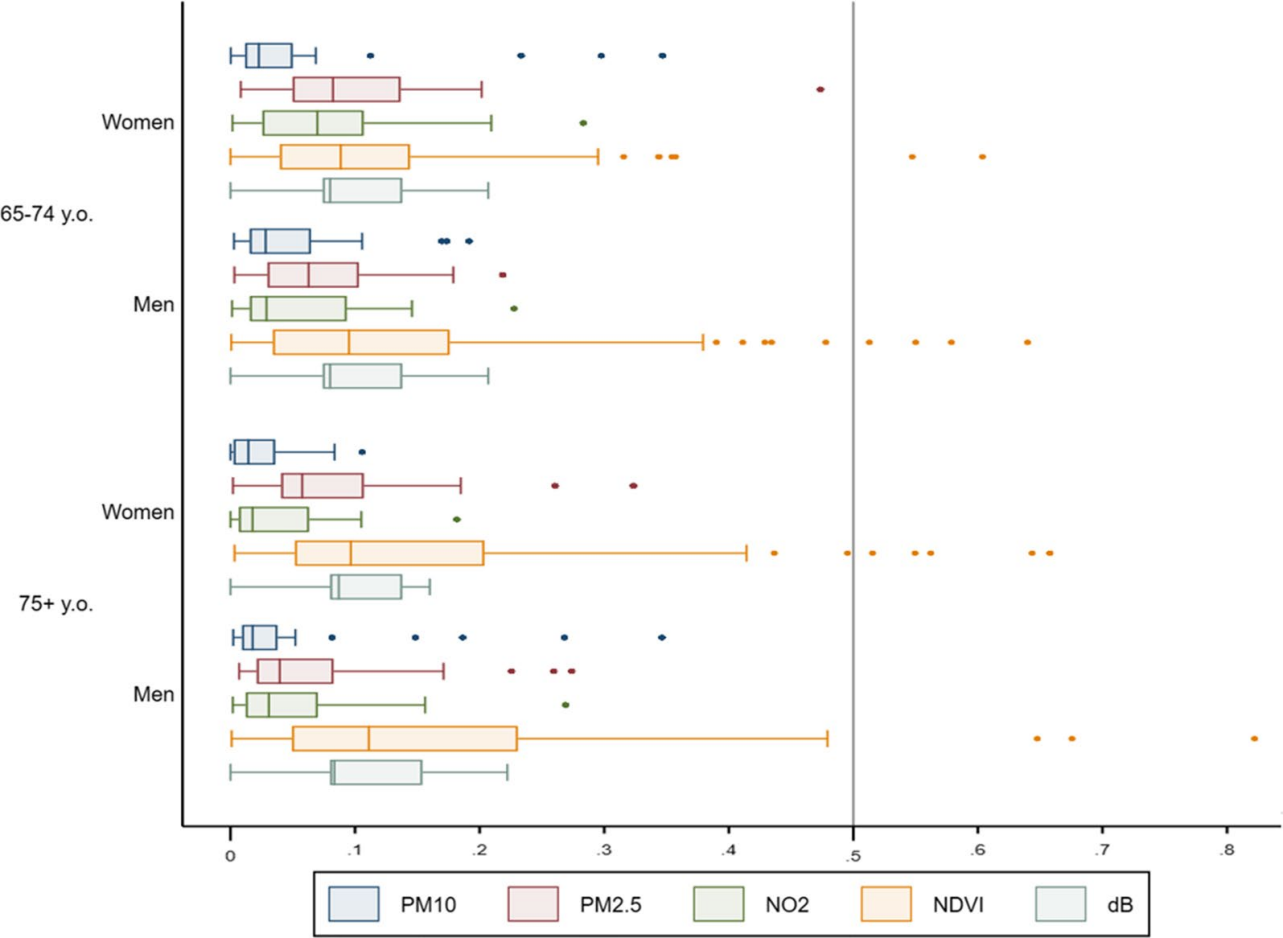
Distribution boxplots for differences between residence and dynamic exposure per the interaction between gender and age

When using multilevel Poisson models to examine the role of individual sociodemographic or contextual variables driving the differences in exposure, we spot significant differences between the types of exposure (Table 3). Model 1 estimates differences in PM10 to be significantly higher for those living in sprawled areas when compared to those living in historic districts and acting as a reference. Model 2 finds no significant association between PM2.5 and any of the introduced variables. Model 3—focused on NO2—finds expected differences in home-dynamic exposure to be 70% higher in those living in sprawl areas when compared to those living in historical districts, once the rest of the covariates are taken into account. Model 4 and Model 5 dedicated to estimating differences in home-dynamic exposures to noise and greenness respectively, did not find any associations between home-based measures of exposure and GPS-track-based dynamic measures of exposure once all the control variables are taken into account.

**Table 3:** Multilevel Poisson models estimating the differences between residential and dynamic environmental exposures among seniors

	Model 1			Model 2			Model 3			Model 4			Model 5		
	PM10			PM2.5			NO2			dB			NDVI		
	Coef.	P > z	95% Conf.	Coef.	P > z	95% Conf.	Coef.	P > z	95% Conf.	Coef.	P > z	95% Conf.	Coef.	P > z	95% Conf.
Gender															
Women	= ref.			= ref.			= ref.			= ref.			= ref.		
Men	0.537	0.163	− 0.22; 1.29	0.279	0.385	− 0.79; 0.31	0.031	0.928	− 0.64; 0.70	0.084	0.735	− 0.40; 0.57	0.098	0.64	− 0.31; 0.51
Urban layout															
Historic district	= ref.						= ref.			= ref.			= ref.		
Sprawl	2.684	0.001	1.07; 4.30	0.643	0.448	− 0.72; 1.75	1.741	0.02	0.28; 3.20	0.386	0.569	− 0.94; 0.71	0.066	0.896	− 0.93; 1.06
Expansion	0.618	0.402	− 0.83;2.06	0.418	0.963	− 0.80; 0.84	0.456	0.447	− 0.72; 1.63	0.355	0.371	− 0.42; 1.13	− 0.194	0.527	− 0.80; 0.41
Income															
High	= ref.						= ref.						= ref.		
Low	− 0.004	0.991	− 0.76; 0.75	0.291	0.851	− 0.63; 0.52	− 0.081	0.819	− 0.77; 0.61	− 0.276	0.297	− 0.80; 0.24	0.226	0.304	− 0.21; 0.66
Age															
64–74 y.o	= ref.						= ref.						= ref.		
75 y.o	− 0.407	0.286	− 1.15; 0.34	0.275	0.911	− 0.57; 0.51	− 0.396	0.247	− 1.07; 0.28	0.133	0.594	− 0.36; 0.62	0.261	0.218	− 0.15; 0.68
N Trips	− 0.017	0.825	− 0.17;0.14	0.056	0.658	− 0.14; 0.08	− 0.006	0.927	− 0.14; 0.13	− 0.001	0.978	− 0.10; 0.09	− 0.05	0.245	− 0.14; 0.03
Cons	− 3.994	0.000	− 5.60; − 2.38	0.48	0.000	− 3.25; − 1.37	− 3.233	0.000	− 4.56; − 1.91	− 2.611	0.000	− 3.51; 1.71	− 1.907	0.000	− 2.61; − 1.21

## Discussion and conclusions

 Our results indicate that significant differences between static and dynamic exposure assessments are only present in selected exposures and would thus suggest that dynamic assessments using GPS-tracking are not providing superior accuracy across all the ranges of exposures.

Regarding air quality—measured using NO2, PM10, and PM2.5—our data suggest that dynamic exposure would only be recommended in the case of the smaller particulate matter (PM2.5) for which a discrepancy of almost 6% between residential and dynamic exposures was detected. This is consistent with previous findings that have found major differences between static and dynamic PM2.5 exposure [21, 89, 90]. Both NO2 and PM10 presented discrepancies below 1.5% leading us to believe that the potential gains in accuracy derived from the use of GPS tracking would not outweigh its challenges and burdens. Our results suggest that among seniors, only studies dealing with PM10 and NO2 exposure among very specific population sub-groups would require a GPS-tracking methodology. An example of that would be studies focused on the older age range (seniors over 75 years old), or in low-income areas, in which discrepancies between static and dynamic exposures are closer to 2%. In the most complete study to date on the differences between static and dynamic measures of traffic-related exposure set in Shenzen, China, Yu et al. [40, 41] found the static measures to overrepresent exposures by almost 30%. Our results confirm Yu’s findings for the specific case of seniors and the major discrepancies found when assessing PM2.5. Our models, however, seem to indicate that in the context of Barcelona static measures are underrepresenting real exposures and not overrepresenting them.

Regarding noise exposure, our estimates suggest that studies focusing on seniors and noise would greatly benefit from dynamic exposure assessment and the use of GPS tracking. According to our data, static home-based measures tended to overestimate noise exposure by 10%, and that overestimation was higher in areas with heavy traffic, such as the example or historic districts. Interpretation of dynamic vs static measures of sound exposure in the literature is diverse. While Kou et al. [91] argue that mobility constrained groups such as seniors might find more difficulties in adjusting their behavior to the presence of high noise pollution, others such as Ma et al. [89, 90] have argued that residents of high-noise pollution areas might be self-selecting their mobility routes and destinations to travel to less noisy areas which may lower their average dynamic-exposure. In that specific case, the fact that seniors don’t have mobility patterns that are strictly fixed by the presence of the workplace would contribute to their flexibility to self-select for less noisy areas.

Finally, studies interested in the exposure of seniors to greenness and NDVI, would not seem to benefit from dynamic exposures in urban areas similar to Barcelona. In part, these results might be explained by the high density of curbside street trees in Barcelona [79]. Linear methods calculated around pedestrian tracks might be positively affected by the large presence of curbside trees. In contrast, because Barcelona has a lack of parks and open green spaces, home exposure to NDVI might also be limited to curbside trees. Previous studies have also failed to find a significant difference between dynamic and static exposures to greenness [92]. Differences in terms of NDVI exposure in Barcelona seem highly dependent on the residential location, suggesting that in this case, it might be worth considering the kind of urban area that is being studied before deciding on whether to use dynamic exposures. In the specific case of greenness and seniors, home-based exposure is even more important given the links between home-based measures of greenness and mental health [67].

Overall, our study failed to uncover large discrepancies between static and dynamic exposures. This might be explained by several factors. First, the study population—seniors above 65 years old—are also one of the population groups that tend to use their local neighborhood the most, with travel patterns that are usually concentrated around their home residence and with activity spaces that cover fewer distances away from home [29]. Taking most trips in the vicinities of home would likely attenuate the expected accuracy benefits from a dynamic exposure approach. This would confirm the previous hypothesis that states that daily exposures estimates obtained from the two approaches only differ substantially if an individual’s time spent away from home is large [21, 93–95]. These findings, supported by the study by Yu et al. [40, 41] in China suggest that dynamic exposure assessment may only be warranted when studying those population groups that tend to spend more time in out-of-home activities in nonresidential neighborhoods and are thus exposed to considerable different conditions over the day.

In practical terms, the fact that most seniors have lower mobility rates makes them potentially more vulnerable to the conditions of their local neighborhoods. As such, seniors with higher mobility ranges may be able to select for the less polluted environment during their daily everyday mobility, while seniors with more limited mobility capacities may find it difficult to lower their exposures to hazardous local conditions. In the specific case of Barcelona however, previous research has demonstrated that the seniors increased use of proximity and the local neighborhood is a matter of preference and potential of the built environment [28, 96] rather than a case of spatial entrapment caused by limited mobility options [97].

The specific characteristics of the local built environment in Barcelona may also contribute to explaining the general low accuracy gains from dynamic exposures, as low variance in the built environment characteristics has also been known to affect other similar study settings [98]. Studies dealing with more diverse urban environments might accrue additional gains from dynamic exposure, suggesting that the decision on whether or not to use a tracking methodology and dynamic measures would depend not only on the population group that is being studied but also be location dependent.

In the interpretation of results a couple of limitations need to be considered. For once, mobility data and exposure data came from different sources as participants were not equipped with specific devices to capture exposure while they were moving. Thus, the opportunistic use of city council exposure data creates a time-gap between the moment on which the tracking took place (2017) and the exposure data (2018). This may create small accuracy inconsistencies that need to be considered, however in recent years Barcelona has failed to produce a significant change in the levels of emissions, noise or greenness [99, 100] and that makes us confident that baseline conditions did not change significantly between 2017 and 2018. Secondly, the use of NDVI as a measure of greenness of exposure differs in nature to the other measures of exposure as NDVI in not a physical measure but a simplified indicator which is highly sensor dependent. This could affect future replicability of the study’s findings.

This study is the first to assess the need for dynamic exposure assessment when studying exposures of seniors. In this case, we use seniors as an example of a population group with low mobility levels among which using dynamic measures of exposure would not accrue significant accuracy gains in environmental exposure assessments. This qualifies the often extended idea that dynamic measures of exposure are greatly needed across all kinds of studies [101] and would point to the need to adapt the use of dynamic vs static measures of exposure to the kind of mobility patterns of the population group of interest. Generally, studies on environmental exposures need to consider human mobility and spatial variations of exposures if they want to avoid misinterpretations. Accurate estimates of exposures are key for policymakers to identify spatial or social inequities in exposures and to design interventions that can alleviate them. The search for accuracy cannot hide, however, the need to use more efficient and cost-effective methods for each research question.

## Supplementary Information

**Additional file 1: Table S1.** Bi-variate correlations between static and dynamic measures of exposure.

## Data Availability

The datasets used and/or analyzed during the current study are available from the corresponding author upon reasonable request.
