# Supplementary material for: Comparison of static and dynamic exposures to air pollution, noise, and greenness among seniors living in compact-city environments
Source: Int J Health Geogr. 2023 Jan 28;22:3. doi: 10.1186/s12942-023-00325-8 (PMC9884423; doi:10.1186/s12942-023-00325-8)
Supplement: Supplementary file 1 — Additional file 1: Table S1. Bi-variate correlations between static and dynamic measures of exposure. [file 12942_2023_325_MOESM1_ESM.docx]

| Table S1: Bi-variate correlations between static and dynamic measures of exposure. | | | | | | | | | | | | |
| --- | --- | --- | --- | --- | --- | --- | --- | --- | --- | --- | --- | --- |
| Static Measures | | | | | |  | Dynamic Measures | | | | | |
|  | NO2 | PM10 | PM2.5 | Noise | NDVI |  |  | NO2 | PM10 | PM2.5 | Noise | NDVI |
| NO2 | 1 | .415^**^ | .437^**^ | .749^**^ | -.543^**^ |  | NO2 | 1 | .480^**^ | .462^**^ | .459^**^ | -.414^**^ |
| PM10 | .415^**^ | 1 | .510^**^ | .465^**^ | -.223^*^ |  | PM10 | .480^**^ | 1 | .538^**^ | .250^**^ | -.274^**^ |
| PM2.5 | .437^**^ | .510^**^ | 1 | .379^**^ | 0.066 |  | PM2.5 | .462^**^ | .538^**^ | 1 | .143^**^ | .079^*^ |
| Noise | .749^**^ | .465^**^ | .379^**^ | 1 | -.404^**^ |  | Noise | .459^**^ | .250^**^ | .143^**^ | 1 | -.407^**^ |
| NDVI | -.543^**^ | -.223^*^ | 0.066 | -.404^**^ | 1 |  | NDVI | -.414^**^ | -.274^**^ | .079^*^ | -.407^**^ | 1 |
|  | **. Correlation is significant at the 0.01 level (2-tailed). *. Correlation is significant at the 0.05 level (2-tailed). | | | | | | | | | | | |
